# Unlikely Nomads: Settlement, Establishment, and Dislodgement Processes of Vegetative Seagrass Fragments

**DOI:** 10.3389/fpls.2018.00160

**Published:** 2018-02-14

**Authors:** Samantha Lai, Siti Maryam Yaakub, Tricia S. M. Poh, Tjeerd J. Bouma, Peter A. Todd

**Affiliations:** ^1^Experimental Marine Ecology Laboratory, Department of Biological Sciences, National University of Singapore, Singapore, Singapore; ^2^Department of Environment and Ecosystems, DHI Water & Environment, Singapore, Singapore; ^3^Department of Estuarine and Delta Systems, Royal Netherlands Institute for Sea Research (NIOZ), and Utrecht University, Yerseke, Netherlands

**Keywords:** dispersal, connectivity, asexual recruitment, flume, movement ecology

## Abstract

The dispersal of seagrasses is important to promoting the resilience and long-term survival of populations. Most of the research on long-distance dispersal to date has focused on sexual propagules while the dispersal of vegetative fragments has been largely overlooked, despite the important role this mechanism might play. In this study, we proposed a conceptual model that categorizes vegetative fragment dispersal into seven fundamental steps: i.e., (i) fragment formation, (ii) transport, (iii) decay, (iv) substrate contact, (v) settlement, (vi) establishment, and (vii) dislodgement. We present two experiments focusing on the final steps of the model from substrate contact to dislodgement in four tropical seagrass species (*Cymodocea rotundata, Halophila ovalis, Halodule uninervis*, and *Thalassia hemprichii*), which are critical for dispersed vegetative fragments to colonize new areas. We first conducted a mesocosm experiment to investigate the effect of fragment age and species on settlement (i.e., remains on the substrate in a rising tide) and subsequently establishment (i.e., rooting in substrate) rates. To determine dislodgement resistance of settled fragments, we also subjected fragments under different burial treatments to wave and currents in a flume. We found that both initial settlement and subsequent establishment rates increased with fragment age. *H. ovalis* was the only species that successfully established within the study period. After settlement, dislodgement resistance depended primarily on burial conditions. Smaller species *H. ovalis* and *H. uninervis* were also able to settle more successfully, and withstand higher bed shear stress before being dislodged, compared to the larger species *T. hemprichii* and *C. rotundata*. However, the ordinal logistic regressions did not reveal relationships between the tested plant morphometrics and the energy needed for dislodgement (with the exception of *C. rotundata*), indicating that there are potentially some untested species-specific traits that enable certain species to withstand dislodgement better. We discuss the implication our findings have on the dispersal potential for different species and the conservation of seagrasses. This study represents the first effort toward generating parameters for a bio-physical model to predict vegetative fragment dispersal.

## Introduction

The study of the movement ecology determining how seagrasses disperse is critical to understanding the exchange of genetic material, and their persistence in changing environments (McMahon et al., 2014; Weatherall et al., 2016). The dispersal of seagrass propagules fundamentally affects the genetic structure and diversity of metapopulations (Kendrick et al., 2012), with larger dispersal ranges creating more opportunities for genetic mixing, leading to greater diversity (Hamrick and Godt, 1996). Over time, genetically diverse meadows are better able to resist disturbances, including stressors related climate change (Reusch et al., 2005; Ehlers et al., 2008). In light of this, elucidating the mechanisms for seagrass dispersal is vital to the management and conservation of seagrass meadows, particularly at the regional scale.

Seagrasses can disperse at several life stages, and the dispersal range at each stage can vary, often by orders of magnitude (McMahon et al., 2014). Seeds and pollen for many species tend to be neutrally or negatively buoyant (Harwell and Orth, 2002; Orth et al., 2006), leading to them being transported over short distances and remaining within the parent meadow (Kendrick et al., 2012). Occasionally, seeds can be dispersed by secondary biotic vectors such as dugongs, fish or waterfowl, for distances ranging from meters to thousands of kilometers (Sumoski and Orth, 2012; McMahon et al., 2014). However, long-distance dispersal has generally been attributed to buoyant fruits or reproductive shoots bearing seeds which, depending on hydrodynamic conditions, can travel up to hundreds of kilometers from the source meadow (Harwell and Orth, 2002; Erftemeijer et al., 2008; van Dijk et al., 2009; Ruiz-Montoya et al., 2012).

Sexual propagules are not the only mechanism for long-distance dispersal. Seagrass fragments, i.e., pieces of rhizome with roots and at least one green leaf shoot (Ewanchuk and Williams, 1996), can produce new shoots and roots following detachment from the parent plant and re-establish elsewhere to create a new independent ramet (Rasheed, 2004). While this mechanism has been used to explain identical genotypes occurring across large distances in multiple cases (Diaz-Almela et al., 2008), there are only a few published studies documenting successful events, possibly because it is difficult to confirm in situ. In Success Bank, Australia, Campbell (2003) reported natural recruitment of *Posidonia australis* fragments, with 69% surviving the 23-month study in sites that were deep and had relatively little wave movement. Di Carlo et al. (2005) documented the re-colonization of *Posidonia oceanica* via vegetative fragments in a backfilled dredged site, which they reasoned provides a stable environment for “entanglement and anchorage” of the fragments. While the literature so far has indicated that successful dispersal and establishment events may be rare (Ewanchuk and Williams, 1996; Rasheed, 2004), this mechanism could be important for meadow connectivity given the large quantities of vegetative fragments that are produced and exported (Diaz-Almela et al., 2008). Ewanchuk and Williams (1996) conservatively estimated that 2–4% of the leaf shoot population in Mission Bay, California, is lost as fragments every year – representing a substantial fragment source. In addition, fragments can be produced year-round, through disturbance events such as storm events, boat propeller damage and dredging (Thayer et al., 1975; Erftemeijer and Robin Lewis, 2006), and are not restricted temporally by seasonality or phenology in the same way sexual propagules are.

**FIGURE 1:**
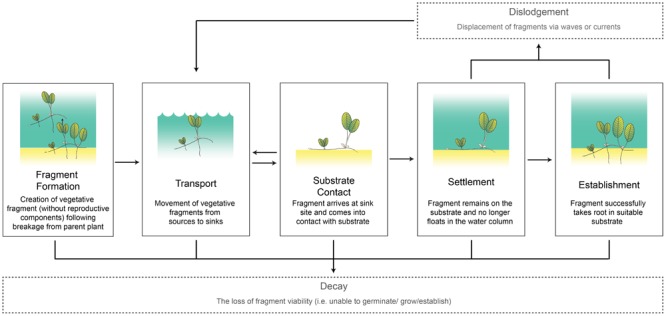
Seagrass fragment transport conceptual model with definitions. Seagrass images adapted from Catherine Collier, Integration and Application Network, University of Maryland Center for Environmental Science (ian.umces.edu/imagelibrary/).

To understand the role of vegetative fragments in promoting connectivity, it is important to identify and investigate the key processes in fragment dispersal. We propose it can be broken down into seven core processes (**Figure 1**). Five of these processes occur in sequence:

(i) Fragment formation: The creation of the vegetative fragment from an existing meadow without reproductive components (e.g., seeds or fruit) following breakage from a parent plant.
(ii) Transport: The movement of the vegetative fragment from source to sink. The distance traveled during this phase largely depends on how long the fragment stays in the water column without decaying and the ambient hydrodynamic conditions.
(iii) Substrate contact: The arrival of the fragment at the sink site and coming into contact with suitable substrate. During this phase, the fragment has the opportunity to settle through either mechanistic (e.g., root hair attachment) or physiological processes (e.g., loss of buoyancy).
(iv) Settlement: The fragment remains on the substrate (no longer floats in the water column).
(v) Establishment: The fragment takes root in the substrate.

The two processes below occur outside this sequence. Dislodgement can occur after Settlement or Establishment stages, while Decay of the fragment can occur at any point.

(vi) Dislodgement: Displacement of fragments from the substrate via ambient hydrodynamic forces.
(vii) Decay: The loss of fragment viability resulting in the inability to establish and/or grow.

A suite of species traits and environmental factors may exert an influence over each of the processes described above. For example, each species has distinct rhizome biomechanical properties, which could form fragments at different rates, leading to varying fragment output. Abiotic factors, such as wave and current energy, substrate type or water column nutrients, that interact with these traits are also potentially important. A number of preliminary studies have been conducted on several species to elucidate how plant traits and environmental factors (e.g., plant morphometrics, source of fragment, season of fragment production) and their interactions affects the success at different stages (see Ewanchuk and Williams, 1996; Hall et al., 2006; Berković et al., 2014; Weatherall et al., 2016), and therefore contribute to overall connectivity of seagrass meadows via the production and transport of their vegetative fragments. However, it is patently clear from the paucity of the scientific literature that there are knowledge gaps on the processes controlling settlement, establishment, and dislodgement of seagrass vegetative fragments, which are key for resolving whether vegetative fragments are a viable conduit for establishment in new areas, and the mechanism by which this can happen.

Our study focuses on the last four stages in fragment dispersal (i.e., substrate contact, settlement, establishment, and dislodgement resistance) in the context of a nearshore intertidal setting. In particular, we focus on how vegetative fragments in tropical seagrass species can settle and establish in nearshore intertidal, soft sediment habitats, because this environment is especially suited for the growth of seagrass meadows (McKenzie et al., 2016). In these habitats, tides are key to initiating fragment contact with substrates, as fragments can remain positively buoyant for several weeks (Harwell and Orth, 2002) and may not be able to settle otherwise. We first investigated whether seagrass fragments that have been transported for varying lengths of time (i.e., fragment age) would settle or establish at different rates under a simulated tidal regime. We also tested settlement frequency of fragments under different burial treatments, and subsequently their dislodgement thresholds under hydrodynamic forcing from both wave and currents in a flume. Sediment movement can be dynamic in the nearshore environment, and the resulting burial from such movement is often perceived as a negative impact on seagrass meadows (Cabaço and Santos, 2007). However, a degree of burial can potentially help fragments resist dislodgement, thereby improving the chances of a fragment persisting post-settlement. These burial events can arise as part of natural processes such as bioturbation from burrowing animals or the motion of sand waves, or anthropogenic disturbances like dredging and beach stabilization (Cabaço et al., 2008). Aside from burial treatments, we also investigated whether plant morphology influenced the amount energy needed to dislodge settled fragments. Infantes et al. (2011) showed that seedlings of the temperate species *P. oceanica* can remain anchored under periodic flows if their root lengths are 0.35 times the square-root of their leaf area, suggesting that plant morphology can also play an important role in fragment dislodgement.

These experiments are, to our knowledge, the first efforts toward generating parameters for a bio-physical model for tropical vegetative fragment dispersal under different conditions commonly encountered in the nearshore environment, allowing for predictions about when and how this process can contribute toward connectivity among seagrass meadows.

## Materials and Methods

### Fragment Settlement and Establishment

Fragments of three tropical seagrass species, *Halophila ovalis* and *Halodule uninervis, Cymodocea rotundata* were collected from Chek Jawa, Singapore (1°24′32.1″N 103°59′32.6″E) and transported in seawater to the St. John’s Island National Marine Laboratory (SJINML) for the mesocosm experiments. To simulate seagrass fragments that have been detached for different lengths of time (which we refer to as the fragment age), the collected fragments were held in aerated flow-through outdoor aquaria for 1 week, 2 weeks, and 3 weeks.

Mesocosms designed to recreate a local semi-diurnal tidal cycle were used in the experiment to simulate a nearshore environment where the positively buoyant fragments can periodically come into contact with suitable substrate for settlement and establishment. Each mesocosm (240 mm × 135 mm × 140 mm, L × W × D) was filled with 10 mm of sediment collected from the local seagrass meadows and was subjected the following tidal regime over 14 days: 4 days with two low-tides a day (at lowest point: 5 mm of seawater above the substrate), 4 days with two mid-tides a day (45 mm of seawater above the substrate), and 6 days without tidal change (100 mm of seawater above the substrate). Each low- or mid-tide lasted for 2.5 h, and occurred at 1000 h and 2200 h. This simulated low-tide provided the opportunity for substrate contact. The tidal simulation was achieved by opening controlled valves (measuring 4 mm in diameter), which were installed at different heights of the mesocosm to allow the water to drain out to the desired tidal height. Throughout the experiment, a slow flow-through (7 ml s^-1^) was maintained from a single reservoir to ensure similar conditions across all the mesocosms, while avoiding movement that may have agitated the sediment or seagrass. The average temperature across mesocosms was 29.7°C (SD ± 0.36), while daylight intensity ranged from 400 to 700 μmol m^-2^ s^-1^. These temperature and light conditions are comparable to those experienced in the field (Yaakub et al., 2014).

Fragments of ages 0 week (i.e., fresh fragments), 1 week, 2 weeks, and 3 weeks from the three species (*n* = 9) were randomly assigned to the mesocosms, with each mesocosm only containing one fragment to ensure independence. Each fragment consisted of three shoots, including the apical meristem, connected with rhizome and roots. The buoyancy status of the fragments – floating, settled, or established – was recorded every 2 days for 14 days. Fragments that remained on the substrate during high-tide were considered to have settled or established. To differentiate between settled and established fragments, the mesocosm was manually agitated. If not established, fragments would move around on top of the substrate when the tank was agitated.

### Fragment Dislodgement Resistance

Seagrass fragments of *H. ovalis, H. uninervis, C. rotundata*, and *Thalassia hemprichii* were collected from Singapore before being transported to Netherlands packed in moist paper towels within 48 h of collection. The fragments were kept in a large aerated aquarium heated to 29°C with a 12-h light regime for the duration of the 2-week experiment.

*Halophila ovalis* and *H. uninervis* fragments (at least two shoots and not longer than 120 mm), and *C. rotundata and T. hemprichii* fragments (at least one shoot and not longer than 100 mm) were transplanted into sand-filled pots (mixture of fine and medium sand) made of PVC pipes of dimensions 150 mm in height and 120 mm in diameter. These pots had moveable bottoms made of 3 mm PVC disks that could be adjusted to alter sediment depth (Balke et al., 2011). The fragments were transplanted under three different treatments that mimicked potential sediment burial scenarios (*n* = 10) (Balke et al., 2011): (i) rhizomes and roots buried 20 mm deep in sediment, (ii) rhizomes and roots buried 5 mm deep in sediment, and (iii) not buried (i.e., just substrate contact). These fragments were emersed for approximately 4 h, mimicking a very long low-tide. This was to ensure that we were testing dislodgement scenarios for fragments that have had the most opportunity (with respect to time) to settle and establish. To determine whether plant morphometrics affect the amount energy needed to dislodge fragments, the number of shoots, maximum leaf width, maximum leaf length, maximum root length, and rhizome length of each fragment were measured prior to transplantation into the pots.

A seawater-filled racetrack flume of 17.5 m long and 0.6 m wide, with a consistent water depth of 320 mm, was used to test the effect of the bed shear stress of currents and waves on the dislodgement of seagrass fragments under different burial treatments (Bouma et al., 2009). The bed shear stress under various current and wave conditions were calculated according to Balke et al. (2011). We first carried out experiments with tidal flow, followed by experiments with waves. Potted fragments were placed into the test section of the flume such that the sediment was flush with the bottom of the flume (similar to Balke et al., 2011). If fragments floated up when submerged, they were considered to have failed to settle. If fragments successfully settled, they were then subject to bed shear stress.

A flow speed of 0.1 m s^-1^ (bed shear stress = 0.01 N m^-2^) was generated in the flume at the start of each trial, and flow speed was increased in steps of 0.1 m s^-1^ every 2 min, to a maximum speed of 0.6 m s^-1^ (bed shear stress = 0.40 N m^-2^). The experiment was only halted when the fragment was dislodged from the sediment. If the fragment was not dislodged at the top speed of 0.6 m s^-1^, we determined their critical erosion, or the amount of sediment that needed to be removed for dislodgement to happen (Balke et al., 2011; Infantes et al., 2011). This was achieved by inserting 3-mm PVC plates under the bottom of the PVC pots, and removing the sediment at the top to ensure it was flush with the flume bottom. The fragments were then subjected to flow speeds of 0.3 m s^-1^ (bed shear stress = 0.10 N m^-2^) for 2 min or until dislodgement. This sediment removal step was repeated until dislodgement.

A second flume experiment was carried out to test the effect of wave action on the dislodgement of the seagrass fragments. In this experiment, two one-burial treatments were tested – burial in 5 mm of sediment, no-burial (i.e., just substrate contact). As with the laminar flow experiment, settled fragments were subjected to wave conditions with maximum bottom shear stresses of 0.15–0.33 N m^-2^ in 0.03 N m^-2^ increments every 2 min. If no dislodgement occurred at the maximum bottom shear stress of 0.33 N m^-2^, we determined the critical vertical erosion using the 3-mm PVC plates. Per sediment removal step, the fragments were subjected to a maximum bed shear stress of 0.24 N m^-2^ for 2 min or until the fragments dislodged. Both wave and current experiments are summarized in **Table 1**.

**Table 1:** Summary of experimental procedures on the effect of laminar flow and waves on seagrass dislodgement.

Experiment	Treatments	Protocol
Effect of laminar flow (current) on dislodgement	(A) Rhizomes and roots buried 20 mm-deep in sediment. (B) Rhizomes and roots buried 5 mm-deep in sediment. (C) Rhizomes and roots are not buried, just left in contact with substrate.	(1) Submerge potted fragment into flume. (2) Apply flow speeds of 0.1 m s^-1^ for 2 min or until fragment dislodges. (3) If not dislodgement occurs, increase flow in steps of 0.1 m s^-1^ every 2 min. (4) If no dislodgement occurs at maximum speed of 0.6 m s^-1^, add 3-mm PVC plate and remove the displaced sediment at the top. (5) Apply flow speed of 0.3 m s^-1^ for 2 min or until fragment dislodges. (6) If no dislodgement occurs, repeat steps 4 and 5 until dislodgement occurs.
Effect of waves on dislodgement	(A) Rhizomes and roots buried 5 mm-deep in sediment. (B) Rhizomes and roots are not buried, just left in contact with substrate.	(1) Submerge potted fragment into flume. (2) Apply wave to create a maximum bottom shear stress of 0.15 N m^-2^ for 2 min. (3) If not dislodgement occurs, increase waves energy in steps of 0.03 N m^-2^ maximum bed shear stress every 2 min. (4) If no dislodgement occurs at maximum bed shear stress of 0.33 N m^-2^, add 3-mm PVC plate and remove the displaced sediment at the top. (5) Apply waves of maximum bed shear stress 0.24 N m^-2^ for 2 min or until fragment dislodges. (6) If no dislodgement occurs, repeat steps 4 and 5 until dislodgement occurs.

### Statistical Analyses

In the settlement and establishment experiment, Kaplan–Meier survival analyses were used to estimate time taken before fragments settled or established in the sediment, and a log-rank test was used to compare the settlement and establishment between the fragment ages. Similar analyses were performed to compare mortality as well. Analyses were performed in using the *survival* package (Therneau, 2009) in R 2.14.2 (R Core Team, 2015).

In the dislodgement experiment, the proportion of successfully settled fragments after emersion in the no-burial treatment between the four species were tested using a four-sample test for equality of proportions in R (R Core Team, 2015). The mean bed shear stress needed for dislodgement were compared between species and burial treatments separately using a Kruskal–Wallis *H*-test, as the data did not satisfy Cochran’s test (Winer et al., 1971) for homogeneity of variance needed for an ANOVA. As the number of fragments that successfully settled after 4 h of substrate contact were low across all the species, the no-burial treatment was not included in the ANOVA analysis.

As both flume experiments increased bed shear stress in incremental steps and data were zero-inflated and did not conform to a normal distribution, an ordinal logistic regression was applied to elucidate whether any of the morphometric measurements had an effect on bed shear stress needed to dislodge the fragments. The effect of morphometric measurements on the critical erosion were analyzed using general linear models with a Poisson distribution. Model selection was based on Akaike Information Criterion (AIC). Analyses were performed in R 2.14.2 (R Core Team, 2015), with the *ordinal* package used for the ordinal logistic regression (Christensen, 2015).

## Results

### Fragment Settlement and Establishment

Total fragment mortality across the species and treatments was 36.7% over the course of the 14-day mesocosm experiment. The survival analyses revealed that the mortality rate did not differ significantly among species (*p* > 0.05) or fragment ages (*p* > 0.05). Individuals that died prior to establishment or settlement were removed from subsequent analyses.

**FIGURE 2:**
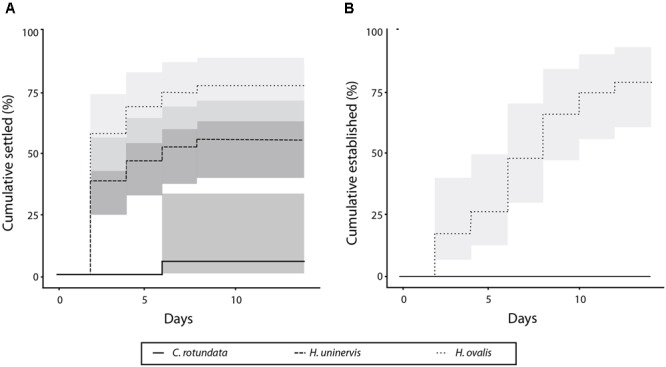
Percent of fragments settled **(A)** and established **(B)** over 14 days for each species.

**FIGURE 3:**
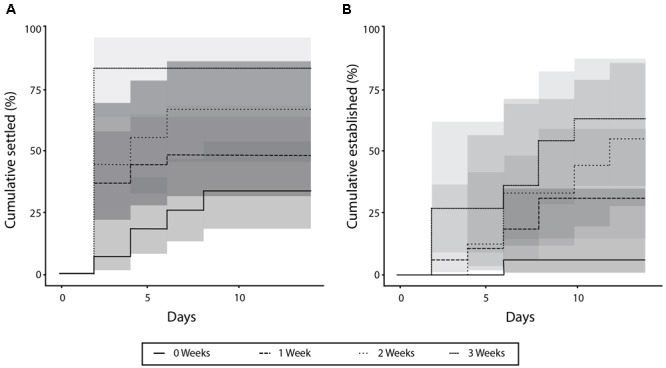
Percent of fragments settled **(A)** and established **(B)** over 14 days for each the four age treatments.

The three species settled at significantly different rates (*p* < 0.01) (**Figure 2A**), with the majority of *Halophila ovalis* fragments (77.7%) settling by the end of the experimental period, compared to 55.5% for *H. uninervis* and only 7.7% for *Cymodocea rotundata*. Of the *H. ovalis* fragments that settled, most (58.3%) did so within the first 2 days during the simulated “low-tides.” None of the *H. uninervis* or *C. rotundata* fragments established by the end of the 14 days, whereas 78.2% of the surviving *H. ovalis* fragments did (**Figure 2B**). Fragments of different ages also settled at significantly different rates (*p* < 0.01) (**Figure 3A**). The settlement rate increased with age, with 83.3% of oldest 3-week-old fragments settling, followed by the 2-week (66.7%), 1-week (48.1%), and fresh fragments (33.3%).

Of the three species, only *H. ovalis* fragments managed to establish within the 14 days. When establishment rates among different aged fragments were compared, a similar trend was evident, with the older (2- and 3-week) fragments all establishing, followed by 83.3% of 1-week fragments and just 20.0% of fresh fragments (*p* < 0.01) (**Figure 3B**). Unlike settlement, establishment happened consistently across the experimental period, and was not affected by the tidal regime.

### Fragment Dislodgement

**FIGURE 4:**
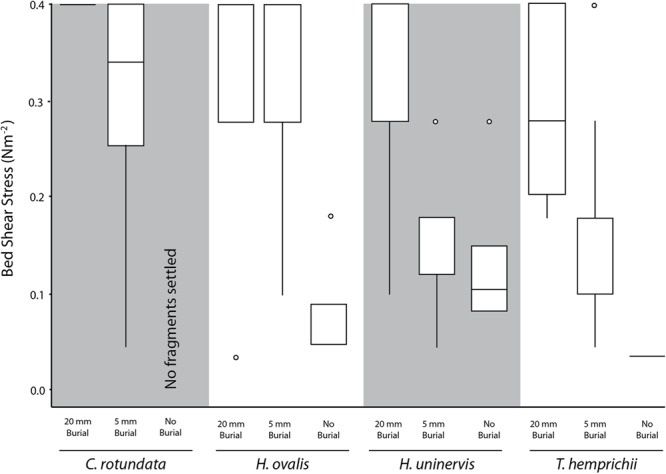
Box plot of the current-generated bed shear stress (N m^-2^) needed to dislodge fragments of different species under each burial treatment. Midline represents 50% quartile, and top and bottom of each box represent 75 and 25% quartiles, respectively.

**FIGURE 5:**
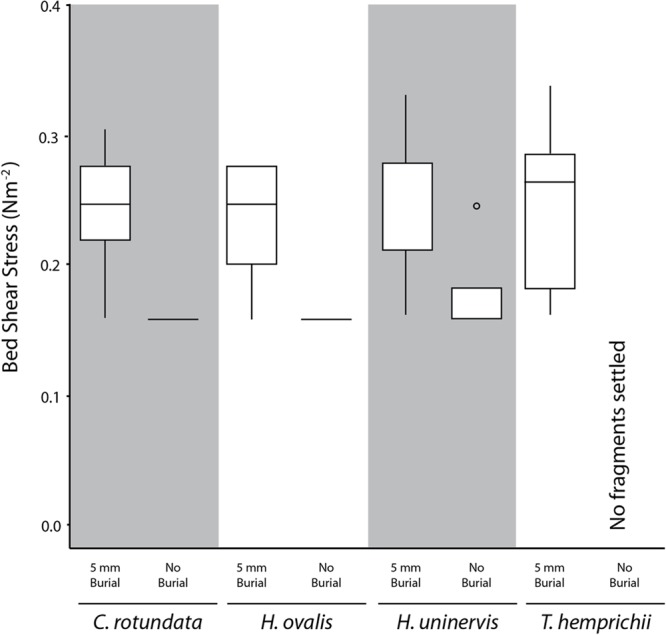
Box plot of the wave-generated bed shear stress (N m^-2^) needed to dislodge fragments of different species under 20 mm burial treatment. Midline represents 50% quartile, and top and bottom of each box represent 75 and 25% quartiles, respectively.

Without burial, only 21.2% of the seagrass fragments that had contact with the substrate for 4 h were not able to settle (i.e., floated up immediately), and thus did not require any dislodgement. A higher proportion of the smaller species managed to settle, with 40% of *H. ovalis* and 32% of *H. uninervis* settling, compared to just 5% in the larger species *T. hemprichii* and *C. rotundata* (χ^2^= 12.27, *df* = 3, *p* < 0.01). On average, the settled *H. ovalis* fragments required 0.07 N m^-2^ (SE ± 0.02, *n* = 6) (**Figure 4**) of current-generated bed shear stress, and 0.16 N m^-2^ (SE ± 0.00, *n* = 3) (**Figure 5**) of wave-generated bed shear stress to be dislodged. The settled *H. uninervis* required slightly higher forces to be dislodged, with 0.12 N m^-2^ (SE ± 0.06, *n* = 4) and 0.18 N m^-2^ (SE ± 0.04, *n* = 4) current-generated and wave-generated bed shear stress, respectively. The mean bed shear stress needed to dislodge the settled *T. hemprichii* and *C. rotundata* fragments were unable to be obtained due to a small sample size.

Based on the flume experiments, we determined that different species required significantly different bed shear stress to be dislodged (*H* = 8.36, *p* < 0.05) under laminar flow. The smaller species *H. ovalis* (0.32 ± 0.02 N m^-2^) and *H. uninervis* (0.31 ± 0.02 N m^-2^) required a higher flow-induced bed shear stress than the larger species *C. rotundata* (0.25 ± 0.02 N m^-2^) and *T. hemprichii* (0.24 ± 0.02 N m^-2^) (**Figure 4**). Fragments that were buried deeper also required significantly higher flow-induced bed shear stress to be dislodged (*H* = 31.71, *p* < 0.01). Fragments buried 20 mm in sediment required an average of 0.36 N m^-2^ (SE ± 0.01), compared to 0.21 N m^-2^ (SE ± 0.02) for fragments buried just 5 mm (**Figure 4**).

The ordinal logistic regression failed to reveal any significant relationships between the various morphometrics in each species-treatment combination to the flow-induced bed shear stress needed to dislodge the fragments for *T. hemprichii, H. ovalis*, and *H. uninervis*. For *C. rotundata*, only one variable, the number of shoots had a significant negative correlation with bed shear stress (*p* < 0.05; **Table 2**). Only some fragments in the 20 mm burial treatment could not be dislodged at the highest flow speed, and had to have their critical vertical erosion quantified. There was no significant difference between critical erosions for the different species (*p* > 0.05), and none of the morphometrics measured were correlated with critical vertical erosion.

**Table 2:** Ordinal logistic regression between morphometric measurements of *C. rotundata* and bed shear stress needed to dislodge fragments. The final model selected was *BSS ∼ Longest Shoot+ Number of shoots* with an AIC of 57.2.

	Estimate	Standard Error	*z*-value	Pr (>|*z*|)	
Number of shoots	–1.22	00.61	–2.01	0.04	^∗^
Longest Shoot	–0.22	0.11	–1.94	0.07	NS

*^∗^Indicates *p* < 0.05; NS = not significant.*

When the fragments were dislodged by waves rather than laminar flow, there was no significant difference in the bed shear stress needed among the four species (*H* = 0. 90, *p* > 0.05) (**Figure 5**). Both *H. ovalis* and *H. uninervis* required an average of 0.23 N m^-2^ (SE ± 0.02) and 0.25 N m^-2^ (SE ± 0.02), respectively, to be dislodged, while *C. rotundata* required 0.24 N m^-2^ (SE ± 0.02) and *T. hemprichii* 0.24 N m^-2^ (SE ± 0.02). None of the species morphometrics measured were correlated with wave-induced bed shear stress needed for dislodgement for either of the treatments. All the fragments were also dislodged by the maximum wave-induced bed shear stress, so no critical vertical erosion was recorded.

## Discussion

The mechanisms for vegetative fragment dispersal in seagrasses have long been overlooked despite evidence that it could be important process for connectivity. This study investigates the final four critical stages – substrate contact, settlement, establishment, and dislodgement resistance – of vegetative fragment dispersal. We found that initial settlement and establishment rates increase with fragment age, and that they tend to settle during period of low-tides when they come into contact with the substrate. However, following settlement, establishment events seem to be rare, and were only recorded in one species – *Halophila ovalis –* in our study. Once settled, the morphometrics of the plant is not a major factor in determining the amount of energy needed to dislodge a fragment. Rather, it is dependent on the extent to which the fragment is buried.

### Effect of Age and Species on Settlement and Establishment Rates

Settlement rates are a factor in determining the dispersal potential of a fragment. The faster a fragment settles, the shorter the distance that the fragment can travel before settling (Weatherall et al., 2016), and eventually establishing. Older fragments settled and established quicker, suggesting there may be a window of opportunity during which settlement and establishment is optimal – when the fragment has enough time to float away from the parent meadow, but not too long that it decays, loses viability, and is no longer able to establish. For example, based on local hydrodynamic conditions in the Singapore Straits, which can reach maximum current speeds of 2.5 m s^-1^ [extracted from a series of points in a MIKE 21 Flow Model (DHI, 2009) hydrodynamic module of Singapore], we estimate that it will take a fragment approximately 12 days to reach Tanjung Piai on the western tip of the Malaysian Peninsula, and approximately 7 days to reach Pulau Batam (Batam Island), in the Indonesian Riau Archipelago south of Singapore. Both these transport durations are well within the maximum fragment survivability period, although this should be verified by modeling. We should note that these estimates lie within the same range as modeling simulations made on both coral and giant clam larvae originating from Singapore, which managed to reach the Riau Islands of Indonesia within 12–15 days of spawning (Tay et al., 2012; Neo et al., 2013). While seagrass fragments patently behave differently from larvae, the hydrodynamic potential for long-distance dispersal remains.

Most of the fragments settled during low-tides and establish soon after. Oxygen is continuously lost from roots and rhizomes when in contact with reducing sediment, which could thus lead to a lowering of the buoyancy and facilitate the settlement of fragments onto the substrate (Borum et al., 2007). This study is the first of its kind to re-create the tidal conditions to demonstrate the process that a seagrass fragment would undergo in order to settle and establish itself; a process, which until now, has never been observed or demonstrated. Seagrass fragments tend to remain positively buoyant for more than 2 weeks (Harwell and Orth, 2002), and fragments may often be reliant on external forces such as tides to help them settle. Of the three species tested, *H. ovalis* was the only one to successfully establish within the experimental period. Establishment has only been previously recorded in pioneering-species *H. wrightii, H. johnsonii* (Hall et al., 2006), and climax-species *P. oceanica* (Di Carlo et al., 2005), *P. australis*, and *Posidonia coriacea* (Campbell, 2003). More than half of the *H. uninervis* fragments in our study were able to settle, but *C. rotundata* mostly remained positively buoyant throughout, and it is possible that both these species require a longer time before they are able to establish, if at all. This attribute may enable their fragments to travel further from the parent meadow, but might also reduce their chances of successful establishment due to reduced viability. *H. ovalis* is known to have high reproductive output and is often a colonizer to bare substrate (Rasheed, 2004), and its quick settlement–establishment trait could contribute to its success as a pioneering species, especially in areas of newly accumulated sediment.

Aside from tides, there have been several suggestions by others as to what factors affect settlement and establishment, though few have been tested rigorously. The presence of algae has been raised as a possible facilitator of establishment, by trapping the fragments and helping them stay in contact with the sediment for longer to promote rooting (Hall et al., 2006). Fragments have been observed to settle early in the morning, but would become positively buoyant by mid-morning, suggesting that respiration could be involved in fragments becoming negatively buoyant and settling (Hall et al., 2006). Thomson et al. (2015) recorded that substrate conditions, such as smaller grain size and higher organic content (i.e., factors that enhance the ‘stickiness’ of the sediment), were positively correlated with establishment rates, indicating that sediment characteristics could also be important.

### Effect of Burial Treatments and Plant Morphometrics on Dislodgement

Our study showed that there was a significant difference among species in relation to settling successfully and resisting dislodgement. When the fragments were emersed without burial (i.e., just substrate contact), most the fragments were not able to settle successfully and still floated up in the water column (e.g., incoming tide), particularly for the larger species *C. rotundata* and *T. hemprichii*. The smaller species, *H. uninervis* and *H. ovalis* experienced better success rates at settlement during the tidal window. In fact, these two species also demonstrated resistance to some flow-induced bed shear stress even without burial after 4 h of emersion. These species have finer roots (0.4–0.7 mm) (Connell et al., 1999; Kiswara et al., 2009) relative to *C. rotundata* and *T. hemprichii* (>1.0 mm) (Kiswara et al., 2009), which might have resulted in better adhesion to wet sediment particles due to a larger surface area to mass ratio.

Once settled, a vegetative seagrass fragment needs to withstand being dislodged by currents and waves before it is established (see concept model, **Figure 1**). Our results indicated that growth form was not a crucial factor in determining the amount a force a fragment could withstand. Of the four species examined, only *C. rotundata* had a single morphometric trait – number of shoots – that had a negative relationship with the current-generated bed shear stress. More shoots would have led to larger leaf area, which is known to create drag in the water column leading to less force needed for dislodgement (Infantes et al., 2011). However, this trend was not observed in the other species. Seagrass leaves are flexible, and this trait is known to help them reduce drag and shear stress (Bouma et al., 2005). In temperate species *Zostera noltii*, leaf flexibility has been shown to actually help reduce hydrodynamic stresses near the bed, thereby reducing erosion (Peralta et al., 2008). It is also possible that there are untested traits that allow the smaller species like *H. ovalis* and *H. uninervis* to withstand higher bed shear stress than the larger *T. hemprichii* and *C. rotundata*. It is difficult to draw substantial conclusions without further experimentation focusing on the drag experienced by species with different leaf areas.

It was clear that, once the fragments were buried, the extent of burial had a stronger effect on the amount of force needed to dislodge the fragments from the substrate. Deeper burial conditions required higher mean current- and wave-induced bed shear stress before fragments could be dislodged. While this result seems intuitive and can be expected, it is important to note that even minimal sediment burial enabled seagrass fragments to resist drag generated at lower current speeds between 0.1 and 0.3 m s^-1^ on seagrass species with varying strap-like leaves. This again demonstrates the importance of burial (even shallow burial of 5 mm) within the context of a seagrass fragment establishing in bare substrate.

### Dispersal Potential vs. Settlement and Establishment Success

The results of the settlement and establishment experiments show that the species that have fragments that can stay afloat the longest (and should disperse the furthest) are not the ones with the highest establishment success. On the one hand, *C. rotundata* fragments do not settle quickly, and therefore have the potential to be transported greater distances from the parent meadow, but require a specific set of conditions (i.e., burial, as well as low current and wave energy) before they can successfully establish at a new site, and might decay before being able to do so. On the other hand, *H. ovalis* fragments not only settle faster, but the fragments of this species can withstand more force (both wave and current) before they are dislodged, even without burial.

The outcomes of our experiments suggest that there is potentially an important role for vegetative seagrass fragments in dispersal and establishment of seagrass in areas with bare suitable substrate, but that a specific set of circumstances must occur in order for this to take place. Settlement is most likely to happen in older vegetative fragments, and at low-tide. However, establishment is a rare event and will likely not happen unless there is burial or some other mechanism of entrapment of the seagrass vegetative fragment into the sediment. This is possible, for example, if there is burial by bioturbators on sandy shores, or where there are existing organisms such as algae in the habitat that could hold the fragment (Hall et al., 2006). Bioturbators have already been shown to facilitate other aspects of aquatic plant ecology such as seed burial (Blackburn and Orth, 2013; Zhu et al., 2016), and accidental burial of vegetative fragments through the reworking of sediment by marine invertebrates would certainly help in the burial process. Even a small degree of burial increases the amount of energy required to dislodge the fragment, the same is likely to be true for entrapment.

### Implications for Seagrass Conservation

Current knowledge regarding the long-distance dispersal of seagrasses via vegetative fragment is relatively limited. This study contributes critical data on the poorly documented settlement–establishment steps of this mechanism – providing the basis for a future bio-physical model to predict the movement of fragments. This model would elucidate how seagrass fragments contribute to population connectivity and help identify source and sink sites, information that is vital for conservation and coastal planning. The model should also evaluate how important this dispersal mechanism is relative to dispersal via sexual propagules. We predict that, when sexual reproduction is limited (either seasonally or due to low fecundity) (Hall et al., 2006), it is likely that dispersal and recruitment via fragmentation will become crucial to range expansion and maintaining genetic diversity (Thomson et al., 2015).

The findings of this study also have applications in the ecological engineering of coastlines. Many seagrass habitats have been lost to coastal modification, but there exists an opportunity to build artificial shores that facilitate the recruitment of seagrasses, thereby creating new meadows without the need for active (and costly) transplantation. For example, unintended recruitment has been recorded in Singapore at a reclaimed shoreline (Yaakub et al., 2014), resulting in the formation of a small (>10 ha) seagrass meadow in the intertidal beach behind a breakwater. We have shown that at least four species of tropical seagrass vegetative fragments, with some degree of burial, are able to withstand hydrodynamic forces that are typical within a shallow coastal embayment, lagoons, and other low-energy nearshore habitats (Lund-Hansen et al., 1997; Warner et al., 2008). Dispersal and establishment by vegetative fragments are especially important for seagrass in areas where the rate of sexual reproduction is low. By designing coastlines to include soft-sediment environments with low hydrodynamic energy, it is possible to maximize windows of opportunity for seagrass fragment establishment, and promote the natural formation of new meadows.

## Author Contributions

SL and SY designed the both experiments, conducted the flume experiment, analyzed the data, and drafted the manuscript. TP designed and conducted the mesocosm experiment, and contributed to manuscript revision. TB and PT contributed to the experimental design and manuscript revision.

## Conflict of Interest Statement

The authors declare that the research was conducted in the absence of any commercial or financial relationships that could be construed as a potential conflict of interest.
